# Is a combination of individual consultations, text message reminders and interaction with a Facebook page more effective than educational sessions for encouraging university students to increase their physical activity levels?

**DOI:** 10.3389/fpubh.2023.1098953

**Published:** 2023-06-28

**Authors:** Eman Alsaleh

**Affiliations:** School of Nursing, Philadelphia University, Amman, Jordan

**Keywords:** physical activity, behavioral intervention, consultations, reminders, facebook, university students

## Abstract

**Background:**

Physical activity (PA) has been consistently reported as a crucial component of disease prevention and improvement of people’s health. Nevertheless, data has evidenced a decline in physical activity levels among adults in Jordan. Although previous behavioral change interventions have documented efficacy in increasing physical activity among adults, the PA levels is low among adults. A new motivational intervention that focuses on changing behavior toward performing the recommended level of physical activity is on need.

**Objective:**

This two-arm single-center randomized controlled trial aimed to measure the efficacy of a multi-component behavioral intervention (including goal setting, self-monitoring, and feed-back) for increasing physical activity levels and self-efficacy for exercise and decreasing body mass index and blood pressure among students at a Jordanian University.

**Setting:**

Philadelphia University in Jordan.

**Methods:**

A behavioral intervention based on individualized consultations, text messages reminders and interaction with a Facebook page was compared with educational sessions in terms of efficacy for increasing physical activity levels among students at Philadelphia University.

**Results:**

The intervention and control groups were comparable at baseline. At 6 months a significant increase was seen in the moderate physical activity and walking levels of the intervention group compared with the control group. The mean change (SD) in total METs of moderate physical activity and walking was 503 (325.20) METs/week in the intervention group and 6 (271.20) METs/week in the control group. The mean change (SD) in steps/day was 3,000 (1,217) steps/day in the intervention group and 876 (1120.23) steps/day in the control group. The difference between mean change of the two groups was very significant at 2,124 (−820 to −563). Self-efficacy for exercise scale significantly increased among the intervention group compared with the control group. In addition, body mass index (BMI) declined from the baseline (Mean: 28.23, SD: 4.82) to 6 months (Mean: 25.36, SD: 5.23) for the intervention group.

**Conclusion:**

Behavioral intervention through multicomponent strategies, alongside the implementation of an advanced communication strategy via phone and social media, is effective for motivating adult students to increase their physical activity levels.

**Clinical trial registration:**

ISRCTN54100536.

## 1. Introduction

Physical activity (PA) has been pervasively acknowledged to be a significant variable in terms of prevention and treatment of numerous health problems and improving cognitive functions and quality of life (1–4). On the basis of these significant health benefits of PA, international recommendations have emphasized that in order to derive the optimal health outcomes from PA, individuals should increase the intensity, frequency and duration of their PA. Guidelines recommend performing 150 to 300 min of moderate intensity PA a week, for instance brisk walking (5).

Regardless of PA’s health benefits and the stipulation of public recommendations for PA levels, PA levels remain low among healthy adult people with a percentage of 31% of adults are physically inactive (6). In contrast with the US and UK, the percentage of physical activity in Jordan remains low. Just 12.5% of Jordanian adults are physically active (7), whereas in the US, 20.7% of people meet the PA guidelines of undertaking 30 min of aerobic PA 5 days per week (8). In the UK, it has been reported that 39% of men and 29% of women attained the government’s recommended PA level of engaging in moderate PA for 30 min on five or most days of the week, as measured via a self-reported PA questionnaire (9).

Contributing factors to low performing of and adherence to PA which are consistently reported in the literature include individual characteristics—for example motivation and self-efficacy—perceived barriers to PA, for instance time and access, in addition to the characteristics of PA behavior, such as PA type, intensity and duration (10). Specifically, the foremost barriers to PA reported by adults include time, access to PA, in addition to schedule-related barriers (11). Accordingly, designing an intervention characterized by its accessibility and applicability for people may prove effective in enhancing PA levels among the adult population. Additionally, it is necessary to implement a PA intervention focusing on strengthening individuals’ motivation and their self-efficacy, in order to increase their PA levels and to facilitate their resolution of specific PA barriers which might facilitate their increased PA levels.

International health policy has emphasized the significance of the workplace as an effective environment for encouraging employers in terms of promoting their health and well-being (12, 13). The workplace is considered an ideal location for delivering health behavior programs for transcending commonly cited obstacles to PA, for example ‘lack of time’, as well as for providing access for employers to encourage their engagement in PA (14). Nevertheless, there remains a requirement for further interventions that identify the effectiveness of programs’ comprehensive components that can enhance PA levels.

Individualized consultation offers efficacy in terms of strengthening people’s PA levels, comprising of elements that aim to enhance PA in accordance with individuals’ specific needs (15–19). However, there is lack of evidence regarding the features of effective consultations in maintaining sustained PA levels among healthy people.

Previous interventions that aimed to alter behavior towards healthy lifestyles, such as physical activity, weight reduction and healthy dietary patterns, have showed efficacy of behavioral change strategies in changing behavior. These techniques include goal setting self-monitoring and regular feedback (20–22). Nevertheless, maintenance of a healthy lifestyle as a form of long-term behavior is infrequently reported with regard to these interventions (23). Accordingly, an effective intervention approach comprising a healthy behavioral maintenance program is necessary.

Goal setting is a self-regulation skill encouraging individuals to establish specific behavioral change objectives that fulfil their requirements. It involves details pertaining to individuals’ planning, for example definition of a specific behavior, frequency, intensity or duration, as well as determining context (where, when, how, or with whom they perform the behavior) (24). For instance, “I will walk briskly to my job area for 30 min, 5 days/week for 1 month” (25). Feedback is a form of behavioral change strategy that has evidenced success in reaching the goals that individuals have set, when it is combined with goal setting to alter unhealthy behaviors, for instance physical inactivity and an unhealthy diet (26). Primarily, feedback comprises of information regarding health behavior’s quantity and quality, typically being provided by individuals such as healthcare providers (27). Additionally, it may include providing encouragement to individuals to attain their aims, through telling them that they are able to perform a specific behavior (28). The effective method for providing feedback is via regularly contacting individuals using phone calls, emails and face-to face visits (28). Self-monitoring is another behavioral change strategy that could be implemented for encouraging subjects to observe their health behavior based on their goal-setting over time (29).

Healthy behavior interventions that include increasing physical activity and improving healthy diet must aim to motivate people’s regular engagement in physical activity. Furthermore, it is necessary to enhance people’s access to physical activity programs, which may be successfully implemented via telecommunications, including telephone consultation, mobile phone text messaging and social media.

Significantly, web-based interventions have been developed and evaluated for delivering PA programs. These offer certain advantages over printed materials, face-to-face and telephone contact, for example their accessibility to a significant number of people at low cost, as well as their availability 24 h a day for providing instant and tailored feedback (30). Nevertheless, recent reviews have indicated that such interventions have a limited effect on PA levels, alongside certain methodological limitations such as subjective measurement of PA (31, 32). Consequently, there is a requirement to conduct PA-based interventions that provide an effective and objective method for measuring PA.

Changing behavior has been emphasized by Social Cognitive Theory (SCT) which is considered as one of the most predominant theoretical approaches in PA interventions because it provides a framework for understanding and improving PA (33–35). This theory includes health determinants that identify how health behavior works and that lead to improve health behaviors. The determinants of SCT were tested and applied in previous PA interventions among older people and CHD patients with successful results in increasing PA levels (36–39). The theory focuses on the likelihood that individuals will modify and construct an environment to fit their objectives (27). It describes human behavior as a product of interacting personal, behavioral and environmental domains, which are considered behavioral determinants that influence people and create each individual’s unique behavior (40). A set of determinants to improve health behavior were postulated by this theory that include: (a) knowledge of an individual’s health risks and the benefits of healthy practices; (b) the health goals people set and plan and the strategies for attaining them; (c) perceived facilitators and barriers to healthy behavior; and (d) perceived self-efficacy (belief of personal self-efficacy) (40). The evidence of the success of SCT in these previous studies in increasing PA levels to a high level encourages the use of SCT as the theoretical framework of this intervention.

### 1.1. The study objectives

This research’s primary aim is to investigate the effect of a multi-component behavioral intervention (including goal setting, self-monitoring and feed-back) in terms of increasing physical activity levels among students adults at Jordanian Universities. The secondary aims were to assess the behavioral intervention’s effectiveness for reducing body mass index (BMI) and blood pressure, in addition to enhancing self-efficacy during physical activity. To the best of our knowledge, this research offers the first investigation of behavioral intervention delivered to enhance PA levels among healthy adults in Jordan.

## 2. Methods

### 2.1. Study design

This was a randomized controlled trial, and parallel group study (allocation ratio1:1). Consolidated Standards of Reporting Trials (CONSORT) statement guidelines were adopted in conducting and reporting the trial. Ethical approval for the study was granted in Jordan by the institutional review board at Philadelphia University. All procedures performed in this study involving human participants were in accordance with the ethical standards of the institutional and national research committee and with the 1964 Helsinki declaration and its later amendments or comparable ethical standards.

### 2.2. Participants

The target population were students across all subjects at Philadelphia University in Jordan with no medical illness that prevented them from undertaking moderate physical activity, for example brisk walking. Sample size calculations was based on statistical power and the difference in mean change of PA level (steps per week) between control and intervention group. The calculation was made to detect improvements in PA level of 10,000 steps per day which was considered as a moderate difference between the control and intervention group. Two-sided 5% significance level and power of 80% was estimated. The total sample size of 156 participants was required accounting for up to 15% loss to follow-up.

### 2.3. Procedure

**Figure 1:**
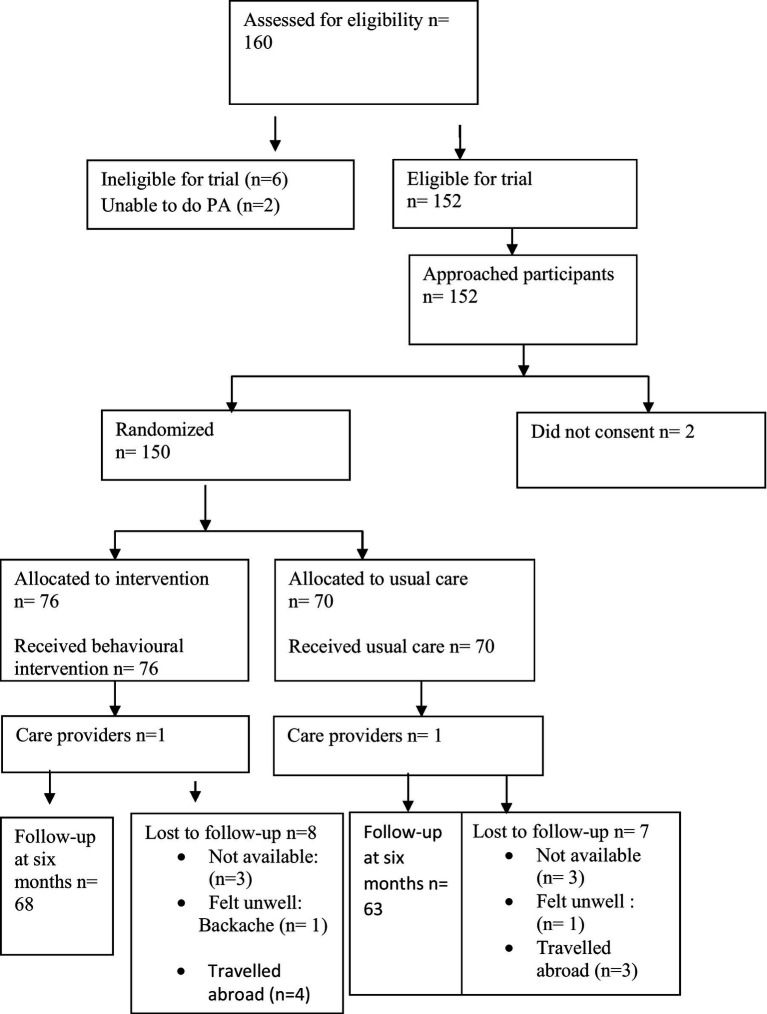
Flow of participants through the study period.

Potential participants were identified by the researcher by meeting them in different times during their gathering in student’s hall at University. Once eligibility was confirmed, the researcher approached the participants in the university and provided them with study information sheets containing contact details for the researcher. Interested participants then contacted the researcher who arranged a suitable time to take informed consent at the University, following which they were recruited into the study and baseline data collected. Recruited participants were randomized to one of two groups. Randomization was undertaken using prepared opaque sealed envelopes, by a researcher who was not involved in recruitment or the delivery of the intervention. Recruitment and baseline data collection took place between February and April 2017. Outcome data were collected at two time points: baseline (immediately after recruitment) and immediately post-intervention (6 months after randomization; Figure 1).

The study was a two-group randomized controlled trial (n = 150). A behavioral intervention based on individualized consultations, reminder text messages and Facebook was compared with educational sessions, in terms of the effectiveness of increasing physical activity levels among healthy adults students at Philadelphia University. Having collected baseline data, the patients were randomly allocated to the two groups (the control and intervention group) through a process of opening opaque, sealed sequence envelopes according to the participant’s numbers, which contained the group name. The educational sessions was given to the control group and comprised of lectures covering subjects pertaining to PA’s health advantage, risks from physical inactivity, as well as methods for increasing PA among students. These lectures were given as part of educational sessions on regular basis (one 60 min lecture every month) which include power point presentation about healthy life aspects such as managing of diet, body weight, stress and PA. The intervention was delivered to the intervention group between February and August 2017 by the main researcher (Eman Alsaleh). This to ensure the consistence and fidelity of the intervention to all participants (with respect to length and duration of contacts and extent of advice given). The participants and the researcher who provided the intervention were not blinded to the intervention because the nature of the intervention should be obvious and interactive between them. However, the data analysis were blinded to the data assessors by entering data on the SPSS to the first and second group without knowing groups allocation to the control or intervention group.

The intervention involved implementing individualized consultations on a regular basis, namely a single face-to-face individualized consultation with the researcher as well as six telephone call consultations (one call per month). The consultations aimed to analyse the current PA status for every individual, enhance the awareness of PA’s advantages, identify and improve the facilitators of PA, diminish barriers to PA, facilitate social support, as well as strengthen self-efficacy. The tailored consultation sought to assist participants with integrating a moderate level of physical activity into their daily routine, for example through performing brisk walking for 30 min daily for a minimum of 5 days per week, or 10,000 steps or more daily, using a bracelet pedometer to count their steps.

These consultations were augmented with motivational SMS text messages reminders (one SMS weekly) sent to participants via their mobile phones and on the Facebook page (Let us Walk) over the 6-month period. The types of messages sent included; (a) reminding participants to attain their goals through increasing their PA to the required level; (b) encouraging participants to resolve their PA obstacles through providing them with examples about ways to overcome barriers to PA; (c) encouraging participants to maintain PA over a long period; (d) strengthening participants’ intentions to undertake PA based on theory of planned behavior (TPB); this included statements to enhance participants’ attitudes, subjective norms and perceived behavior control.

Social cognitive theory (40, 41) was adopted during this intervention as a means of improving PA determinants, including: (a) providing knowledge regarding PA behavior; (b) enhancing perceived facilitators and resolving perceived barriers to PA; (c) implementing self-regulation skills (goal setting and self-monitoring); (d) improving self-efficacy. Self-efficacy theory was applied through enhancing its four sources, namely performance accomplishment, verbal encouragement, vicarious experiences and physiological and emotional status capabilities. These sources are significant for enhancing students’ self-efficacy so as to strengthen their PA levels.

### 2.4. Measurement

#### 2.4.1. Socio-demographic and health information

Baseline socio-demographic and health information was recorded via a socio-demographic and health characteristics questionnaire, developed by the researcher. The questionnaire included age, gender, family and personal income, as well as living location.

#### 2.4.2. Physical activity level

Physical activity level was measured by recording the mean of the number of steps for the week using pedometer and the mean of walking and moderates PA frequency, duration and intensity using international physical activity questionnaire (IPAQ).

The pedometer consists of a bracelet worn around a participant’s wrist during the day. This bracelet pedometer measured individuals’ daily steps. The international recommendation is for 10,000-steps daily, in order to fulfil international recommendations to engage in 30 min of brisk walking daily. This amount of daily steps is considered reasonable estimate of daily activity for apparently healthy adults. Based on currently available evidence, it is proposed the following preliminary indices be used to classify pedometer-determined physical activity in healthy adults (42).

1. <5,000 steps/day is used as a ‘sedentary lifestyle index
2. 5,000–7,499 steps/day is typical of daily activity excluding sports/exercise and it is considered ‘low active’
3. 7,500–9,999 likely includes some volitional activities (and/or elevated occupational activity demands) and it is considered ‘somewhat active
4. >or = 10,000 steps/day indicates the point that should be used to classify individuals as ‘active’.
5. Individuals who take >12,500 steps/day are likely to be classified as ‘highly active’.

The IPAQ asked the individuals to report their level of PA by writing the frequency (days per week) and duration (minutes per day) of each type of PA. To measure the intensity of PA, metabolic equivalents (METs) were calculated. The levels of two types of PA (moderate PA and walking) including frequency (days per week), duration (minutes per day) and intensity (METs) were measured in the intervention of this study.

IPAQ defines walking as including any form of walking from place to place which equals 3.3 METs. Moderate PA is defined as that which needs moderate physical effort and causes some shortness of breath such as carrying light loads, bicycling at a regular pace or doubles tennis. This equals 4 METs. The METs for each type of PA is found by multiplying duration (in minutes) and frequency (in days) by 3.3 for walking and by 4 for moderate PA. The total METs equals the summation of the walking METs and moderate level of PA METs.^1^ The participants were classified as physically active when they met the PA guidelines of performing 30 min of moderate intensity PA 5 days a week (150 min per week), or a combination of walking and moderate-intensity activities achieving a minimum of at least 600 METs-minutes/week.

#### 2.4.3. Blood pressure

The researchers measured blood pressure using an automated electronic blood pressure monitor. To ensure consistency with this device, the participants’ blood pressures were measured using the same BP monitor at baseline as well as at 6 months. Blood pressure was measured in accordance with the criteria for measuring blood pressure as determined by the American Heart Association (43), the British Hypertension Society (44), as well as the Medicines and Health care products Regulatory Agency (45).

#### 2.4.4. Body mass index

Body mass index was measured based on the following equation: [body weight (K gm)/height ( $m^{2}$ ; where m is meter)], by measuring weight during the two data collection points and measuring height at the baseline data collection point. Body weight was measured using the same measure at the two data collection points.

#### 2.4.5. Exercise self-efficacy

Exercise self-efficacy was analysed according to the exercise self-efficacy scale (ESES), which was devised and evidenced as being valid and reliable by Resnick and Jenkins (46). The questionnaire asked the participants to respond to nine items describing situations in which people might experience challenges with engaging in regular PA. The answer per item ranged between 0 (not confident) and 10 (very confident) to rate the present expectation regarding their abilities to undertake moderate PA. The total score was determined by summing the numerical rating for each answer and dividing it by the number of answers. The ESES questionnaire is only available in an English language version, therefore it was translated into an Arabic version by a translator, then checked for validity and reliability. Validity of the ESES was conducted by consulting one cardiac clinic nurse and one cardiologist in Jordan to check for completeness, clarity and readability of the questionnaires with consideration of the Arabic language. The expert people assessed the questionnaire as suitable to use without any corrections. In addition, the internal consistency of the ESES was assessed to ensure the reliability of the questionnaire by conducting a pilot study among 20 adults (both genders, with different educational levels and their ages ranged between 30 and 60 years). The Cronbach’s alpha was 0.90 (*n* = 22) which was very similar to the English version of the scale which was 0.92 (*n* = 187) (46).

#### 2.4.6. Data analysis

SPSS for Window version 20.0 was used to undertake the data analysis. Independent t-test was conducted for measuring the mean difference of the average changes in PA levels between the control and intervention groups. PA level changes among both the intervention and control groups were calculated through subtracting the baseline PA level scores from those at the 6-month follow-up. Intention to treat analysis (ITT) was adopted, including multiple imputation and last observation carried forward. Independent t-test and chi-squared test were applied as a means of comparing the demographic profiles of the intervention and the control group at the baseline, as well as responders with non-responders at the 6-month follow-up point.

## 3. Results

The total number of participants who were randomly allocated to the control group (*n* = 70) and intervention group (*n* = 76) were 146. Sixty-eight of the intervention participants and 68 of the control group completed the outcomes data at the 6-month point. At the baseline, no significant difference was evidenced between the control and intervention groups regarding sociodemographic characteristics (Table 1).

**Table 1:** Sociodemographic characteristics of the participants.

		Control (*n* = 70)	Intervention (*n* = 76)	*P*
Age	Gender	21.2 + −2.3	21.3 + −2.4	0.38
	Male	34 (49)	35 (46)	0.88
	Female	37(51)	39 (54)	
Family income/month	<300	8 (12)	6 (8)	0.47
	301–500	22 (31)	23 (30)	
	501–700	23 (33)	26 (34)	
	>701	17 (24)	21 (28)	
Personal income/month	>50 JD	17 (24.2)	18 (23.7)	0.25
	51–100 JD	34 (48.6)	35 (46)	
	101–150	16 (22.9)	18 (23.7)	
	>150	3(4.3)	5 (6.6)	
Living area	Village	18 (25.7)	19 (25)	0.36
	City	52 (74.3)	57 (75)	

### 3.1. Adherence to behavioral change strategies

Seventy one (93%) of the intervention participants accomplished a minimum of one goal in their PA diary after 3 months of the intervention, while 72 (95%) completed a minimum of one goal 6 months after the intervention. Adherence to the completion of physical activity diaries increased by 5% throughout the intervention period.

### 3.2. Physical activity status

Participants’ physical activity status was determined at the intervention’s outset and conclusion, in accordance with the IPAQ guidelines for classification of PA status. Participants were recognized as having a physically active status when they met the PA guidelines of 30 min of moderate intensity activity 5 days a week (150 min per week), or engaged in a combination of walking and moderate-intensity activities amounting to a minimum of at least 600 METs-minutes/week. Approximately one-third of the sample met the physical activity recommendation at the baseline (intervention: *n* = 24, 32%; control: *n* = 22, 31%). By the conclusion of the intervention, the number of physically active participants had increased significantly among the intervention group from baseline to six months (*n* = 55, 81%), yet had declined in the control group (*n* = 20, 32%; Table 2).

**Table 2:** Physical activity status among study groups.

PA status	Control group		Intervention group	
	Baseline (*n* = 70)	6 Months (*n* = 63)	Baseline (*n* = 76)	6 Months (*n* = 68)
Physically active	22 (31%)	20 (32%)	24 (32%)	55 (81%)
Not physically active	48 (69%)	43 (68%)	52 (68%)	13 (19%)

### 3.3. Physical activity level

Mean scores for moderate PA and walking levels (including frequency, duration and intensity) and METs/week and steps count increased significantly from the baseline to 6-months among the intervention group, although not in the control group. The difference in mean change of walking, moderate PA levels and steps count between the two groups was high (Table 3).

**Table 3:** Change in the physical activity levels by group.

PA levels				Mean (SD)	Mean change (SD)		Mean difference (95% CI)
Moderate PA level	Control Baseline	6 m	Intervention Baseline	6 m	Control group (n = 63)	Intervention group (n = 68)	
Frequency (days/week)	0.80 (1.25)	0.78 (1.35)	0.79 (1.30)1	1 (1.20)	−0.02 (0.62)	0.21 (0.9 7)	0.23 (−0.70 1 to −0.10)*
Duration (minutes/week) Walking	60.2 (22.30)	52.3 (44.4)	58 (22.38)	88 (38.50)	−8 0.1 (22.60)	30 (90.15)	38.1 (−39.98 to −1.58)*
Walking level
Frequency (days/week)	3.44 (1.32)	3.46 (2.30)	3.38 (2.80)	5.52 (5.20)	0.02 (1.83)	2.14 (2.75)	2.12 (−3.57 to −2.00)*
Duration (minutes/week)	110.1 (125)	111.3 101.20	108.3 (140.30)	185.2 (211.24)	1.2 (195.93)	76.9 (124.47)	75.7 (−179.96 to −73.76)*
METs/week	460 (412.20)	466 (415)	462 (422.20)	965 (524.23)	6.00 (271.20)	503 (325.20)	497 (366.01 to 447.80)*
Steps/day	6,992	7,868	6,996	9,996	876 (1120.23)	3,000 (1217)	2,124 (−820 to −563)

The analysis was based on *n* = 63 in the control group and *n* = 68 in the intervention group.

SD, standard deviation; PA, physical activity; METs, metabolic equivalents.

* significant difference when *p* < 0.05.

The mean of moderate PA frequency increased in the intervention group from 0.79 days/week at baseline to 1.0 days/week at 6 months, and in the control group it decreased from 0.80 days/week at baseline to 0.78 days/week at 6 months. The mean change in moderate PA frequency was 0.21 days/week in the intervention group and − 0.02 days/week in the control group. The difference in the mean change between the two groups was 0.23 days/week (95% CI −0.70 to −0.10). The mean of moderate PA duration increased in the intervention group from 0.58 min/week at baseline to 80 min/week at 6 months, and it decreased in the control group from 60.2 min/week at baseline to 52.3 min/week at 6 months. The mean change in moderate PA duration was 30 min /week in the intervention group and − 8.1 min/week in the control group. The difference in the mean change between the two groups was 38.1 min/week (95% CI −39.98 to −1.58).

The mean of walking frequency increased in the intervention group from 3.38 days/week at baseline to 5.52 days/week at 6 months, and in the control group it increased from 3.44 days/week at baseline to 3.46 days/week at 6 months. The mean change in moderate PA frequency was 2.14 days/week in the intervention group and 0.02 days/week in the control group. The difference in the mean change between the two groups was 2.14 days/week (95% CI −3.57 to −2.00). The mean of moderate PA duration increased in the intervention group from 108.3 min/week at baseline to 185.2 min/week at 6 months, and it increased in the control group from 110.1 min/week at baseline to 111.3 min/week at 6 months. The mean change in moderate PA duration was 76.9 min /week in the intervention group and 1.2 min/week in the control group. The difference in the mean change between the two groups was 75.7 min/week (95% CI −179.96to −73.76).

The mean of total PA intensity among the intervention group was 462 METs/week at baseline and this increased greatly to 965 METs/week at the 6-month follow-up point, while in the control group it was 460 METs/week at baseline and 466 at 6-month follow-up. The mean change in total METs was 503 METs/week in the intervention group and 6 METs/week in the control group. This represents a very high difference between the mean changes of the two study groups at −497 METs /week (95% CI 366.01 to 447.80).

The mean of steps/day among the intervention group increased significantly from 6,996 steps/day at baseline to 9,996 steps/day at 6-month follow-up point, while in the control group it was 6,992 steps/day at baseline and 7,868 steps/week at 6-month follow-up. The mean change in steps/day was 3,000steps/day in the intervention group and 876 steps/day in the control group. The difference between mean change of the two groups was very significant at 2,124 (−820 to −563).

### 3.4. Exercise self-efficacy

The exercise self-efficacy scale increased from the baseline to 6-months for the intervention group, yet it did not for the control group. The difference in mean change of exercise self-efficacy between the two groups was high. Mean change (SD) and mean difference (95% confidence intervals) are presented in Table 4.

**Table 4:** Changes in the secondary outcomes by group.

Secondary outcomes		Mean (SD)		Mean (SD)	Mean change (SD)		Mean difference (95% CI)
	Control baseline	6 m	Intervention baseline	6 m	Control group (*n* = 63)	Intervention group (*n* = 68)	
Exercise self-efficacy	5.05 (1.52)	5.35 (1.23)	4.80 (1.18)	8.90 (1.20)	0.30 (0.28)	4.10 (1.50)	3.8 (−2.82 to-1.30)*
SBP	124.23 (12.20)	123.86 (13.23)	124.65 (14.02)	124.12 (11.23)	−0.37 (22.60)	−0.53 (90.15)	−0.16 (3.23 to 10.20)
DBP	74.52 (6.32)	73.68 (7.23)	73.66 (5.04)	74.10 (7.85)	−0.84 (6.30)	0.44 (5.20)	1.28 (2.36 to 10.23)
Body weight	66.23	65.89	66.20	60.52	−0.34	−5.68 (8.23)	6.02 (3.25 to 8.36)*
BMI	28.23 (5.32)	28.24 (5.21)	28.23 (4.82)	25.36 (5.23)	0.10 (4.02)	−2.87 (3.41)	−2.97 (1.56 to 2.02)*

The analysis was based on *n* = 63 in the control group and *n* = 68 in the intervention group.

### 3.5. Blood pressure

No significant difference was apparent in the mean change of SBP and DBP between the two groups, as indicated in Table 4.

### 3.6. Body weight and body mass index

Body weight and body mass index (BMI) declined from the baseline to 6-months for the intervention group, yet it did not do so for the control group, as presented in Table 4.

## 4. Discussion

The objective of this study was to examine the effect of a multi methods (including individualized consultation, text messages reminders and face book interaction) based on behavioral intervention on increasing PA levels among students adults at Jordanian Universities. The secondary aims were to assess the behavioral intervention’s effectiveness for reducing body mass index (BMI) and blood pressure, in addition to enhancing self-efficacy during physical activity. This physical activity-based behavioral intervention was successful in improving PA levels among adults, as well as assisting them with reaching the internationally recommended levels of daily PA, which they benefit from in terms of improvements to their health and the prevention of disease risk factors (47, 48). This intervention includes effective strategies that have resulted in successful increases in physical activity among adult students. Nevertheless, few previous multi-behavior change interventions have explicitly targeted PA improvements. Despite it not having been determined which behavioral strategy offers the greatest efficacy in terms of increasing PA levels through this intervention, using multiple behavioral strategies (goal setting, feedback and self-monitoring) might assist students with enhancing their physical activity levels. In contrast numerous previous behavioral interventions among healthy adults only applied one or two behavioral strategies (21, 22).

The characteristics of goal setting adopted by the participants in this intervention were underpinned by theory-based outcomes, which facilitated participants’ successful establishment of specific, personalized attainable goals, as well as the self-efficacy to attain them. In previous PA interventions among adults, there has been insufficient application of specific goals (49). Providing regular follow-ups with participants via telephone calls (once monthly), text messages (one SMS per week) and Facebook also has a significant effect in terms of motivating participants to increase their physical activity levels and resolve obstacles to PA. Evidence shows that providing individuals with regular follow-ups on their behavioral change progress via phone calls, emails and face-to face visits has greater effectiveness in terms of changing unhealthy behavior compared with providing no feedback at all (28).

The self-monitoring strategy involved using a pedometer bracelet, which is an effective method for motivating students’ strengthening of their PA levels. This is consistent with previous interventions among healthy adults and college students (50, 51). Additionally, participants use of diaries was promoted as a means of recording their PA levels, which could motivate them to improve their PA levels. It is apparent that this method has not been applied during previous interventions among healthy adults.

The individualized consultations delivered as part of this intervention included progress feedback, in addition to tailored advice for participants with regards to establishing their own goals, as well as addressing their personal barriers to exercise. Studies involving adult participants have similarly evidenced how individualized, personalized consultations delivering behavioral change strategies may successfully enhance physical activity levels (52, 53). Furthermore, this research used text messaging as a mechanism for reminding participants about their commitment to regularly engage in PA, as well as to remind them of the strategies that had been discussed to transcend their PA obstacles. The extant literature has documented that using text messages for reminders to attain goals enhances the mechanism via which implementation intention alters behavior, through enhancing the accessibility of plans (54).

Theory-based intervention is a significant variable linked to an intervention’s success. The theory guides the intervention through applying health behavior determinants. Evidence shows that insufficient knowledge concerning PA’s significance in preventing the occurrence of diseases, as well as lack of knowledge of the guidelines pertaining to recommended PA levels, is linked to a lack of PA among adults (55). Thus, providing knowledge regarding the health benefits and guidelines linked to PA levels through this intervention was an effective variable associated with participants’ increased PA levels. Perceived barriers to PA is a common determinant of PA participation among healthy adults and university students (56, 57). Significantly, comprehending the significance of goal attainment is essential for implementing effective goal setting (58), thus having information about PA enabled the participants to effectively perform their set goals and subsequently improve their PA levels.

Enhancing patients’ self-efficacy via goal setting was implemented through teaching the participants to establish attainable goals, beginning with small behavioral changes. Such goals can contribute to strengthening participants’ confidence in engaging in PA, leading them to implement further significant changes through establishing more challenging goals. This is confirmed by participants who reported during the telephone calls that they had initiated change through setting small goals, including a short duration of PA which enabled them to set more challenging goals, including a longer duration of PA. This accords with previous findings that attaining goals strengthens self-efficacy, which subsequently stimulates the establishment of higher goals.

This intervention’s effect in terms of decreasing participants’ body weight and BMI is consistent with extant literature results. Those students who decreased their body weight in this research might improve their health through mitigating the detrimental consequences of weight gain, with regards to the development of risk factors linked to the incidence of chronic diseases such as hypertension, diabetes mellitus and coronary heart diseases. It has been established that obesity has a major negative effect on health due to its contribution to the occurrence of diseases through numerous risk factors such as hypertension, hyperlipidemia, insulin resistance, clotting abnormalities and depression (59–61).

This intervention is characterized by its applicability and accessibility to participants. It comprises of strengthening physical activity levels via participation in regular walking, which increases individuals’ step counts as a method that may be straightforwardly accessed and applied by the students. Through following the goal setting strategy, participants can engage in walking activities by integrating them into their routine activity, for example when travelling to university or doing specific exercises. One review reported that workplace counseling has an effect on increasing people’s PA through encouraging the behavior of walking to work [active travel, (14)]. Accordingly, this intervention’s applicability seems positive in terms of its adoption as PA program, motivating students to integrate the recommended level of PA through their travel to university.

Interestingly, male students showed higher PA levels than female students. Differences in PA levels between genders has been widely studied and research constantly proposed males participate have higher levels of PA compared to females (62, 63). Barriers that may explain gender differences have been reported in previous literature and they include time limitations and being too busy due to caring for family (63). Moreover, the students who live in villages had shown higher steps count than students who live in cities. This finding could be related to the nature of village that characterized with spaces and attractive views that encourages students to perform outdoor PA in contrast to cities that lack of these features. Interventions that aim to increase PA should consider these differences in levels of PA among these groups of students to alleviate their specific barriers to perform PA.

This study cannot confirm which strategy of this multi components based intervention is the main influencer in increasing PA levels, such as telephone consultations, text messages reminders and behavioral change strategies (goal setting, self-monitoring, feedback). Future studies may investigate the most significant behavioral change strategy which increases PA levels to apply it in PA based behavioral interventions. However, increased self-efficacy of participants is important outcome as the intervention aimed to improve participant’s self-efficacy for exercise to help them maintaining PA behavior over time. This emphasizes the success of applying multi components based intervention.

It is significant to evaluate this intervention’s efficacy in terms of its ability to uphold participants’ long-term physical activity, however, the participants expressed no willingness for further follow-up in the form of contact via telephone and text messages, due to their travelling and study issues. Measuring PA interventions’ efficacy over the long term is a challenging step in the research area (64, 65) thus it requires more effective interventions as a means of overcoming the barriers to participant follow-ups over the long term. Moreover, this research was limited by including a specific population from one University, because the research purpose was for the principal researcher to follow-up the participants. Recruiting a generalized population from other Jordanian universities is a crucial approach that must be considered in order to incorporate a representative sample of all students attending Jordanian Universities.

## 5. Conclusion

This multi-behavioral intervention evidenced greater PA levels and self-efficacy for exercise, alongside a decrease in body weight and BMI, among Philadelphia University students. Consequently, this intervention may be implemented as a principal program or in combination with other programs as a means of improving Jordanian university students’ healthy lifestyles. The intervention comprised of multiple behavioral change strategies, with no firm conclusions establishing which intervention elements were responsible for the most success, or whether all elements of the intervention are required at the delivered intensity in order to generate an equivalent magnitude of change. Therefore, further research is necessary to establish the ‘amount’ of intervention required to generate PA behavioral change. This study’s participants were contacted through follow-up appointments only at 6 months, therefore it is not understood whether such behavioral changes are sustained over the long term. Moreover, the intervention’s cost-effectiveness must be ascertained to determine the intervention’s applicability.

## Data availability statement

The original contributions presented in the study are included in the article/supplementary material, further inquiries can be directed to the corresponding author.

## Ethics statement

The studies involving human participants were reviewed and approved by The institutional review board at Philadelphia University/Jordan. The patients/participants provided their written informed consent to participate in this study. Written informed consent was obtained from the individual(s) for the publication of any potentially identifiable images or data included in this article.

## Author contributions

The author confirms being the sole contributor of this work and has approved it for publication.

## Conflict of interest

The author declares that the research was conducted in the absence of any commercial or financial relationships that could be construed as a potential conflict of interest.

## Publisher’s note

All claims expressed in this article are solely those of the authors and do not necessarily represent those of their affiliated organizations, or those of the publisher, the editors and the reviewers. Any product that may be evaluated in this article, or claim that may be made by its manufacturer, is not guaranteed or endorsed by the publisher.

## Footnotes

^1^ http://www.ipaq.ki.se/ipaq.htm
